# BRAF V600E protect from cell death via inhibition of the mitochondrial permeability transition in papillary and anaplastic thyroid cancers

**DOI:** 10.1111/jcmm.17443

**Published:** 2022-06-24

**Authors:** Yanyan Gao, Deyu Zhang, Fei Wang, Dejiu Zhang, Peifeng Li, Kun Wang

**Affiliations:** ^1^ Institute for Translational Medicine, The Affiliated Hospital of Qingdao University, College of Medicine Qingdao University Qingdao China; ^2^ Key Laboratory of Nuclear Medicine, Ministry of Health, Jiangsu Key Laboratory of Molecular Nuclear Medicine Jiangsu Institute of Nuclear Medicine Wuxi China

**Keywords:** anaplastic thyroid cancer cell, BRAF V600E, cell death, mitochondrial permeability transition, papillary thyroid cancer cell

## Abstract

BRAF T1799A mutation is the most common genetic variation in thyroid cancer, resulting in the production of BRAF V600E mutant protein reported to make cells resistant to apoptosis. However, the mechanism by which BRAF V600E regulates cell death remains unknown. We constructed BRAF V600E overexpression and knockdown 8505C and BCPAP papillary and anaplastic thyroid cancer cell to investigate regulatory mechanism of BRAF V600E in cell death induced by staurosporine (STS). Induced BRAF V600E expression attenuated STS‐induced papillary and anaplastic thyroid cancer death, while BRAF V600E knockdown aggravated it. TMRM and calcein‐AM staining showed that opening of the mitochondrial permeability transition pore (mPTP) during STS‐induced cell death could be significantly inhibited by BRAF V600E. Moreover, our study demonstrated that BRAF V600E constitutively activates mitochondrial ERK (mERK) to inhibit GSK‐3‐dependent CypD phosphorylation, thereby making BRAF V600E mutant tumour cells more resistant to mPTP opening. In the mitochondria of BRAF V600E mutant cells, there was an interaction between ERK1/2 and GSKa/ß, while upon BRAF V600E knockdown, interaction of GSKa/ß to ERK was decreased significantly. These results show that in thyroid cancer, BRAF V600E regulates the mitochondrial permeability transition through the pERK‐pGSK‐CypD pathway to resist death, providing new intervention targets for BRAF V600E mutant tumours.

## 1 INTRODUCTION

Thyroid cancer is one of the most common endocrine malignancies in the clinic. In recent years, the incidence rate of thyroid cancer has increased. The BRAF T1799A mutation is a common gene mutation in thyroid cancer, melanoma and colon cancer^1^, ^2^, ^3^, ^4^; Molecular studies have found the BRAF T1799A mutation in approximately 45% of PTC and 25% of apparently PTC‐derived anaplastic thyroid cancers, but not in follicular thyroid cancer (FTC) and benign thyroid tumours.^5^ BRAF T1799A mutation contribute to poor clinicopathologic outcomes of PTC, such as increased extrathyroidal invasion, lymph node metastasis, advanced tumour stage and tumour recurrence.^6^Importantly, BRAF T1799A mutation is often associated with loss of I‐131 activity and recurrence of PTC.^5^ The BRAF V600E mutant has increased kinase activity, resulting in increased phosphorylation of MAPK kinase (MEK) 1/2, which phosphorylates ERK1/2, ERK1/2 activates nuclear transcription factors related to cell growth, differentiation and survival.^4^ Due to the application of molecular markers and analysis in clinical care, some inhibitors targeting molecular markers are in clinical research.^7^

Sorafenib is the first generation of RAF inhibitors, but its clinical efficacy is limited, and it cannot completely inhibit the recurrence of advanced tumours with a high incidence of BRAF mutations, such as melanoma and papillary thyroid cancer (PTC).^8^, ^9^ In addition, MEK inhibitors for the treatment of advanced cancers have not yet achieved good clinical results.^10^, ^11^ It has been reported that MEK inhibitors cannot inhibit the S phase of some tumour cells expressing BRAF V600E.^4^ It has also been found that RAF inhibitors can promote abnormal activation of CRAF.^12^, ^13^ Therefore, the clinical treatment defects of these inhibitors for BRAF V600E positive cancer need to be further studied. The study of other regulatory mechanisms of BRAF V600E will help to develop better drugs to treat BRAF V600E positive cancer.

Many studies have shown that cancer cells carrying the BRAF V600E mutation can resist apoptosis, and the anti‐apoptosis effect caused by BRAF V600E may be a factor affecting drug resistance.^14^, ^15^ Lee et al described the interaction of BRAF V600E with the mitochondria via a mutation‐specific mitochondrial localization.^15^Treatment with sorafenib, the MEK inhibitor U0126 and the BRAF‐specific inhibitor PLX4720 did not change the mitochondrial localization or anti‐apoptotic activity of BRAF V600E in thyroid cancer.^15^ The interaction between BRAF V600E and mitochondrial proteins and the specific molecular mechanisms underlying BRAF V600E‐mediated resistance to mitochondrial apoptosis need to be further studied.

Our results further confirmed that BRAF V600E is located in the mitochondria and resists cell apoptosis by regulating mPTP channel closure. BRAF V600E, located in the mitochondria, increased the levels of p‐ERK/ERK in the mitochondria. Mitochondrial localization of p‐ERK further phosphorylated glycogen synthase kinase‐3β (GSK‐3β), which significantly increased the level of p‐GSK/GSK. Furthermore, we report that mitochondrial ERK is constitutively activated by mitochondrial localization BRAF V600E, which inhibits GSK‐3‐dependent phosphorylation of mitochondrial chaperone cyclophilin D(CypD), making it more difficult for BRAF V600E mutant papillary and anaplastic thyroid cancer cells to open mPTP, so that they are less prone to cell death.

## 2 MATERIALS AND METHODS

### 2.1 Cell culture and treatment

We obtained the undifferentiated thyroid carcinoma cell line 8505C (BRAF V600E) and papillary thyroid carcinoma cell lines BCPAP (BRAF V600E), Nthy‐ori‐3.1 thyroid cell from the German Collection of Microorganisms and Cell Cultures (Braunschweig, Germany). 8505C thyroid cancer cell line was cultured in DMEM supplemented with 10% foetal bovine serum (FBS), 100 U/ml penicillin and 100 μg/L streptomycin in a humidified atmosphere containing 5% CO_2_ at 37°C. BCPAP thyroid cancer cell line was cultured in RIMI‐1640 supplemented with 10% foetal bovine serum (FBS), 100 U/ml penicillin and 100 μg/L streptomycin in a humidified atmosphere containing 5% CO_2_ at 37°C.

### 2.2 Cell death assays

The level of cell death were analysed by PI staining. First, 8505C cells climbing to the carry sheet glass were washed with PBS once after STS (MCE, AM‐2282) (200 nM) treatment. Then the cells were treated with PI (10 mg/ml) on ice for 5 min and washed three times with cold PBS. Next, the cells were fixed on ice with 40% paraformaldehyde for 30 min and washed once with ice cold PBS. Finally, cells were stained with DAPI and the level of cell death was detected with a Nikon Eclipse Ti‐S fluorescence microscope.

### 2.3 Lactate dehydrogenase activity assay

8505C cells were seeded in a 24‐well plate. The cells were treated with empty vector adenovirus or BRAF V600E overexpression adenovirus for 6 h and then the culture medium was changed. After 48 h, the cells were treated with STS (100 nM). LDH activity was measured according to the instructions of a spectrophotometric kit (Nanjing Jiancheng).

### 2.4 TMRM staining

8505C cells were seeded into a 35 ‐mm glass bottom culture dish. After infecting with adenoviruses overexpressing BRAF V600E for 48 h, 8505C cells were treated with STS (200 nM). The cells were incubated for 60 min with 150 nM TMRM (Abmole, M9542) at 37°C. Leica TCS SP8 MP laser scanning confocal microscope was used to obtain sequential cellular fluorescence images.

### 2.5 Calcein‐AM staining

8505C cells were seeded into a 35‐ mm glass bottom culture dish. The cells were resuspended in PBS and loaded with calcein AM staining solution (Beyotime Biotechnology, NO. C2009S) and 3 × CoCl_2_ in the dark at 37°C for 30–45 min. At the end of incubation, culture medium was replaced with medium preheated to 37°C, and the dishes were incubated at 37°C for 30 min in order to ensure that calcein AM was hydrolyzed by lactamase to produce green fluorescent calcein. The cells were washed with PBS 2–3 times, and the fluorescence image of mitochondrial calcein were acquired using a Leica TCS SP8 MP laser scanning confocal microscope.

### 2.6 Immunoblotting

The cells were collected and lysed in cold RIPA buffer containing a mixture of protease inhibitor cocktail (APExBio, No. K1007) on ice for 30 min. The cells were then centrifuged at 13,000 *g* at 4°C for 15 min. The proteins were separated by 12% sodium dodecyl sulphate (SDS)‐polyacrylamide gel electrophoresis and transferred to a PVDF membrane (0.45 μm). TBST (50 mM of Tris, 150 mM of NaCl, 0.01% Tween 20, pH 7.5) containing 5% skim milk powder was used to block the membranes, and the following primary antibodies were probed overnight at 4°C. The primary antibodies used included; GSK‐3α/β (CST, #5676); Phospho‐GSK‐3α/β (CST, #8566); COX IV (CST, #4844); p44/42 MAPK (CST, #4695); Phospho‐p44/42 MAPK (CST, #4370); β‐tubulin (CST, #86298); DYKDDDDK Tag (CST, #8146); β‐actin (Santa Cruz, sc‐47778); Rabbit anti‐VDAC2 polyclonal antibody(absin, abs131498); Calnexin (Santa Cruz, sc‐70481). After washing with TBST three times, HRP‐conjugated secondary antibodies were incubated at 25°C for 1.5 h. After washing with TBST for three times, the membrane was incubated in ECL (Vazyme, E412‐02) for 1–2 min, and the results were visualized with a chemiluminescence analyser. Protein quantification was analysed using Image J software (NIH).

### 2.7 Mitochondrial isolation

8505C cells were homogenized with mitochondrial isolation buffer (250 mM of sucrose, 10 mM of Tris–HCl, pH 7.4, 1 mM of EDTA, 1 mM of PMSF). The homogenate was first placed on ice for 10 min. Afterwards, the cells were repeatedly blown with a 2 mL syringe with a No. 25 needle 10 times, then centrifuged at 600 × *g* at 4°C for 5 min. The supernatant was then centrifuged at 7000 × *g* at 4°C for 10 min. The supernatant contained the cytoplasm, while the precipitates consisted of crude mitochondria. The crude mitochondria were then resuspended in 1 ml mitochondria isolation buffer and centrifuged at 7000 × *g* at 4°C for 10 min, and the supernatant was discarded. Finally, crude mitochondria were obtained.

Isolation of cytosol, mitochondria were as previously described.^16^

### 2.8 Immunofluorescence staining

8505C cells were grown on coverslips and treated with MitoTracker for 30 min. Then the cells were washed with PBS and fixed in 4% paraformaldehyde for 15 min at room temperature. Cells were incubated with anti‐BRAF (Santa Cruz, sc‐55,522) overnight at 4°C. Then PBS was washed 3 times, and then incubated with secondary antibody at 37°C for 1 h. The cells on the coverslip were mounted and observed by Leica TCS SP8 MP laser scanning confocal microscope.

### 2.9 Immunoprecipitation of mitochondrial proteins

The crude mitochondria were obtained by the above method and lysed in a weak RIPA buffer (EpiZyme, PC103) containing a protease inhibitor cocktail (APExBio, No. K1007).The supernatants were incubated with anti‐p44/42 MAPK (CST, #4695) overnight at 4°C. Next, the cells were incubated with Protein A/G PLUS‐Agarose (Santa Cruz) at 4°C for 3–4 h. The beads were then pelleted at 3000 × *g* for 3 min, washed three times with lysis buffer and boiled in 4× SDS loading buffer for 10 min. Anti‐p44/42 MAPK (CST, #4695) and anti‐GSK‐3α/β (CST, #5676) were used for immunoblotting analysis.

### 2.10 Flow cytometry‐based measurement of apoptosis and necrosis using Annexin‐V‐FITC/PI staining

Flow cytometry‐based measurement of apoptosis and necrosis using Annexin‐V‐FITC/PI staining was performed as previously reported.^17^ In brief, the cells were collected after cell treatment and centrifuged at 1200 rpm for 5 min. For FACS analysis, one tube for each sample without staining was used for gating. Other samples were stained with propidium iodide (PI) or FITC‐Annexin‐V alone or PI and Annexin‐V combined. After incubation at 4°C for 15 min, binding buffer was added and flow cytometric analysis (BD accuri, C6 flow cytometry) was performed (FITC with FL1 detector were filtered at 530/30 nm and PI with FL2 detector were filtered at 585/42 nm).

### 2.11 Flow cytometry‐based measurement of mPTP using TMRM

After cell treatment, the cells were collected and centrifuged at 1200 rpm for 5 min. The resulting cell precipitates were resuspended with 100 μl of PBS. For FACS analysis, one tube for each sample without staining was used for gating. Other samples were stained with TMRM at 37°C for 20 min and flow cytometric analysis (BD accuri, C6 flow cytometry) was performed.

### 2.12 Statistical analysis

All experiments were repeated three times (*n* = 3). Using GRAPHPADPRISM6.02 (GraphPad Software Inc) to analyse statistically all data. The experimental results are expressed as the mean ± SD of the samples. When comparing two groups, Student's *t*‐test was used to evaluate the significant differences, and anova was used for more than two groups.

## 3 RESULTS

### 3.1 STS treatment decreased the level of BRAF protein in BRAF V600E mutant papillary and anaplastic thyroid cancer cells

**FIGURE 1:**
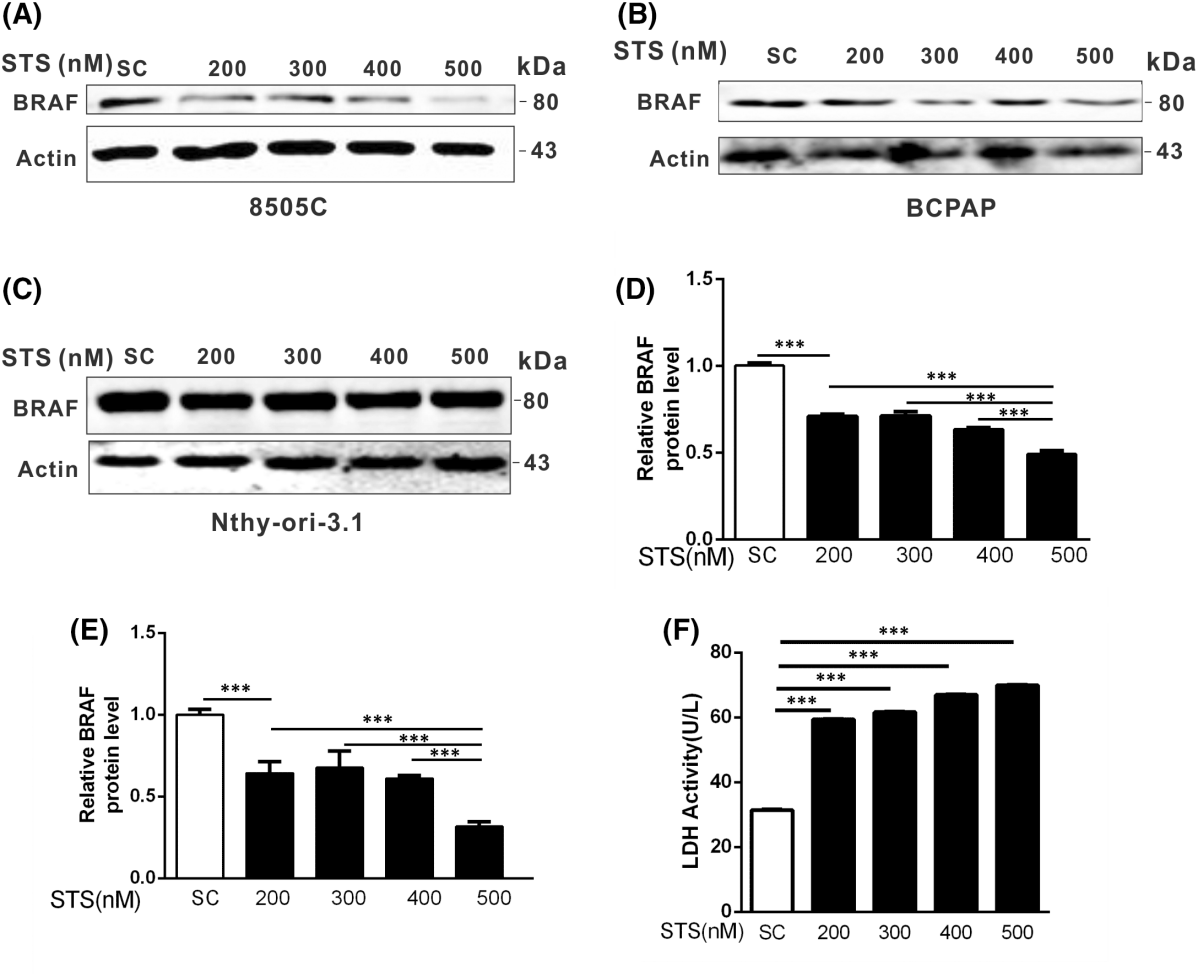
STS treatment decreased the level of BRAF protein in BRAF V600E mutant thyroid cancer cells. (A) 8505C (BRAF V600E), (B) BCPAP (BRAF V600E) cells and (C) Nthy‐ori‐3.1(BRAF WT) cells were treated with STS at different concentrations (200, 300, 400 and 500 nM) for 24 h, the whole cell lysate was extracted and western blotting was performed to detect the protein level of BRAF. β‐actin was used as a loading control. SC, solvent control. Densitometric analysis of the blots in 8505C cells (D) and BCPAP cells (E). Statistical analysis was done by one‐way anova followed by Tukey's multiple comparisons test. *** indicated SC vs. 200 nM and 200 nM, 300 nM, 400 nM vs. 500 nM. (F) After 8505C cells were treated with STS at different concentrations (200, 300, 400 and 500 nM) for 24 h, the LDH activity (U/L) was measured. Statistical analysis was done by one‐way anova followed by Tukey's multiple comparisons test. *** indicated different concentrations (200, 300, 400 and 500 nM) of STS vs. SC

STS is a non‐specific, cytotoxic protein kinase inhibitor isolated from *Streptomyces staurosporeus*.^18^ It can directly or indirectly trigger death pathways, including apoptosis and necrosis, thus leading to cancer cell death.^19^ Lactate dehydrogenase (LDH) with relatively stable enzyme activity can be released into the culture medium from cells caused by apoptosis or necrosis. The degree of cell necrosis can be reflected by detecting the activity of LDH released into the culture after the rupture of cell plasma membrane. The results showed that the LDH level of 8505C cells (BRAF V600E mutant) increased with an increase in STS concentration (Figure 1F). The results showed that 8505C was necrotic after STS treatment. To investigate the potential role of BRAF V600E in cell death, BRAF V600E mutant 8505C and BCPAP papillary and anaplastic thyroid cancer cells were exposed to STS at different concentrations, and the protein levels of BRAF V600E were examined during cell death. We found that the protein levels of BRAF V600E were significantly decreased in BRAF V600E mutant papillary and anaplastic thyroid cancer cells (Figure 1A,B,D,E). In addition, we used Nthy‐ori‐3.1 thyroid cells (BRAF WT) as controls and treated them with STS at different concentrations, and found that the protein levels of BRAF remained unchanged (Figure 1C).These results suggest that BRAF V600E may play an important role in STS‐induced cell death of papillary and anaplastic thyroid cancer cells.

### 3.2 BRAF V600E‐deficient papillary and anaplastic thyroid cancer cells promote cell death

**FIGURE 2:**
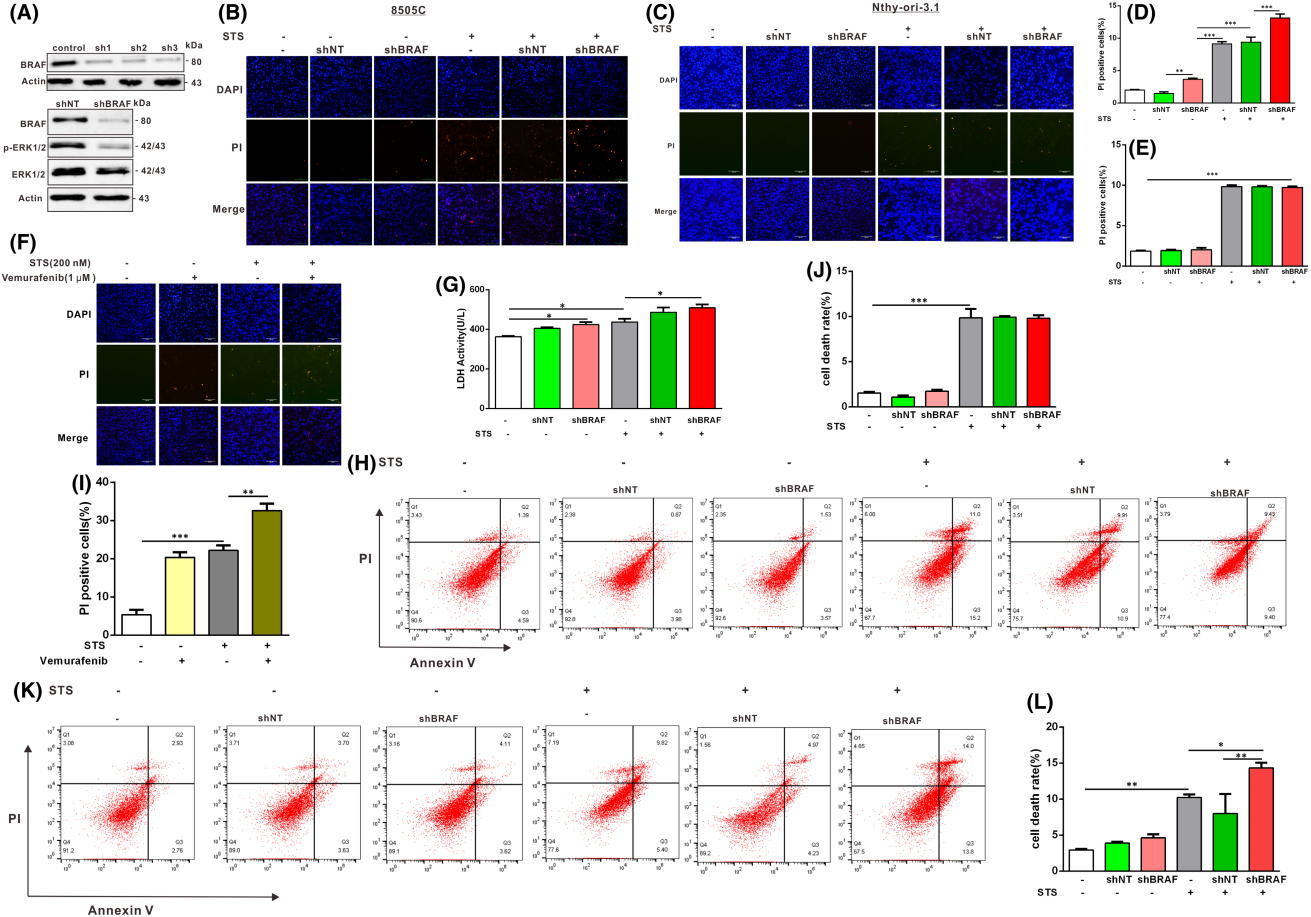
Adenovirus‐mediated knockdown of BRAF V600E in 8505C cells promotes STS‐induced cell death. (A) 8505C cells were infected with adenoviruses expressing BRAF V600E shRNA, and western blotting was used to detect BRAF, phosphorylated and total ERK1/2 levels. β‐actin was used as a loading control. The interference effect was verified by constructing three different BRAF shRNAs constructs. After infection with adenoviruses expressing BRAF shRNA for 48 h, 8505C cells (B) and Nthy‐ori‐3.1 cells (C) were treated with STS (200 nM). These 8505C cells and Nthy‐ori‐3.1 cells were stained with PI (10 mg/ml) and DAPI, and observed under a fluorescence microscope. Scale bar, 200 μm. Two‐way anova followed by Bonferroni multiple comparisons test was performed to statistical analyse the proportion of the PI positive cells in each group of B (D) and C (E). All results are presented as mean ± SD. ** indicated shBRAF vs. shNT. *** indicated control + STS vs. shBRAF and shNT + STS vs. shBRAF + STS or shBRAF. (F) Vemurafenib treatment aggravated STS‐induced cell death in 8505C cells exposed to 200 nM STS for 12 h compared with the control. Cell death was detected by PI assay. Scale bar, 200 μm. (G) 8505C cells were infected with adenoviruses expressing BRAF shRNA and treated with STS (200 nM). The LDH activity (U/L) was subsequently measured. Statistical analysis was done by two‐way anova followed by Bonferroni multiple comparisons test. * indicated control vs. shBRAF. * indicated control + STS vs. control or shBRAF + STS. Nthy‐ori‐3.1 cells (H) and 8505C cells (K) were infected with adenoviruses expressing BRAF shRNA and treated with STS (200 nM). The cell apoptosis and necrosis were assessed by Annexin V‐FITC/PI staining flow cytometry. (I) Two‐way anova followed by Bonferroni multiple comparisons test was performed to statistical analyse the proportion of the PI positive cells in each group of F. All results are presented as mean ± SD. Two‐way anova followed by Bonferroni multiple comparisons test was performed to statistical analyse the cell death rate of Nthy‐ori‐3.1 cells (J) and 8505C cells (L). All results are presented as mean ± SD

**FIGURE 3:**
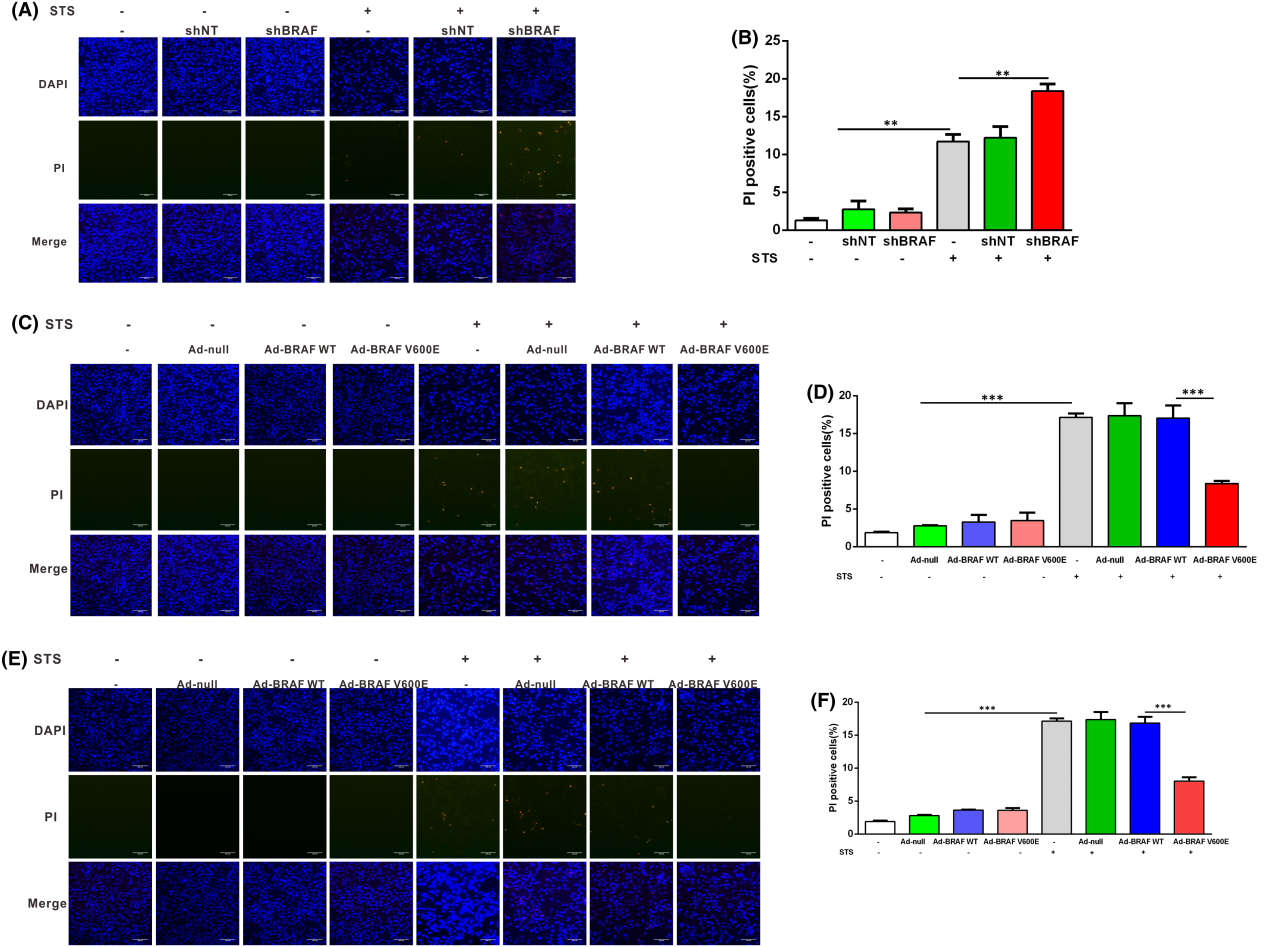
PI staining of BCPAP cells with BRAF V600E overexpression or knockdown. (A) After infection with adenoviruses expressing BRAF shRNA for 48 h, BCPAP cells were treated with STS (200 nM). These BCPAP cells were stained with PI (10 mg/ml) and DAPI, and observed under a fluorescence microscope. Scale bar, 200 μm. (B) Two‐way anova followed by Bonferroni multiple comparisons test performed to statistical analyse of the proportion of the PI positive cells in each group of A. (C) After infecting with adenoviruses overexpressing BRAF V600E and BRAF WT for 48 h, BCPAP cells were treated with STS (200 nM). The BCPAP cells were stained with PI (10 mg/ml) and DAPI, then observed under a fluorescence microscope. Scale bar, 200 μm. (D) Two‐way anova followed by Bonferroni multiple comparisons test performed to statistical analyse of the proportion of the PI positive cells in each group of C. All results are presented as means ± SD. (E) After infecting with adenoviruses overexpressing BRAF V600E and BRAF WT for 48 h, Nthy‐ori‐3.1 cells were treated with STS (200 nM). The Nthy‐ori‐3.1 cells were stained with PI (10 mg/ml) and DAPI, then observed under a fluorescence microscope. Scale bar, 200 μm. (F) Two‐way anova followed by Bonferroni multiple comparisons test performed to statistical analyse of the proportion of the PI positive cells in each group of E. All results are presented as means ± SD

To further confirm the relationship between BRAF V600E and cell death in papillary and anaplastic thyroid cancer cells, we knocked down endogenous BRAF V600E in the BRAF V600E mutant 8505C and BCPAP papillary and anaplastic thyroid cancer cells (Figure 2A). PI staining results showed that knockdown of BRAF V600E sensitized 8505C (Figure 2B,D) and BCPAP (Figure 3A,B) cells to undergo cell death after STS exposure compared to the negative control. However, Nthy‐ori‐3.1 cells that knockdown of BRAF expression showed no significant difference in cell death after STS exposure compared with the control group (Figure 2C,E). To confirm the shBRAF of the cell death effects, we co‐treated selective BRAF inhibitor vemurafenib and found that vemurafenib treatment aggravated STS‐induced death in 8505C cells exposed to STS (Figure 2F,I). Moreover, the results of the LDH assay further showed that STS promoted cellular death of papillary thyroid cancer cells, and the death level of papillary thyroid cancer cells knocked down by BRAF V600E was further aggravated by STS treatment (Figure 2G). The results of flow cytometry‐based measurement of apoptosis and necrosis using Annexin V‐FITC/PI staining further confirmed BRAF V600E‐deficient anaplastic thyroid cancer cells promoted cell death (Figure 2K,L). Annexin V‐FITC/PI staining of Nthy‐ori‐3.1 thyroid cells showed that the down‐regulation of BRAF did not further aggravate the cell necrosis induced by STS (Figure 2J,H). Taken together, these results indicate that BRAF V600E deletion could aggravate papillary and anaplastic thyroid cancer cell death induced by STS exposure.

### 3.3 Overexpression of BRAF V600E attenuates death in papillary and anaplastic thyroid cancer cells exposed to STS

**FIGURE 4:**
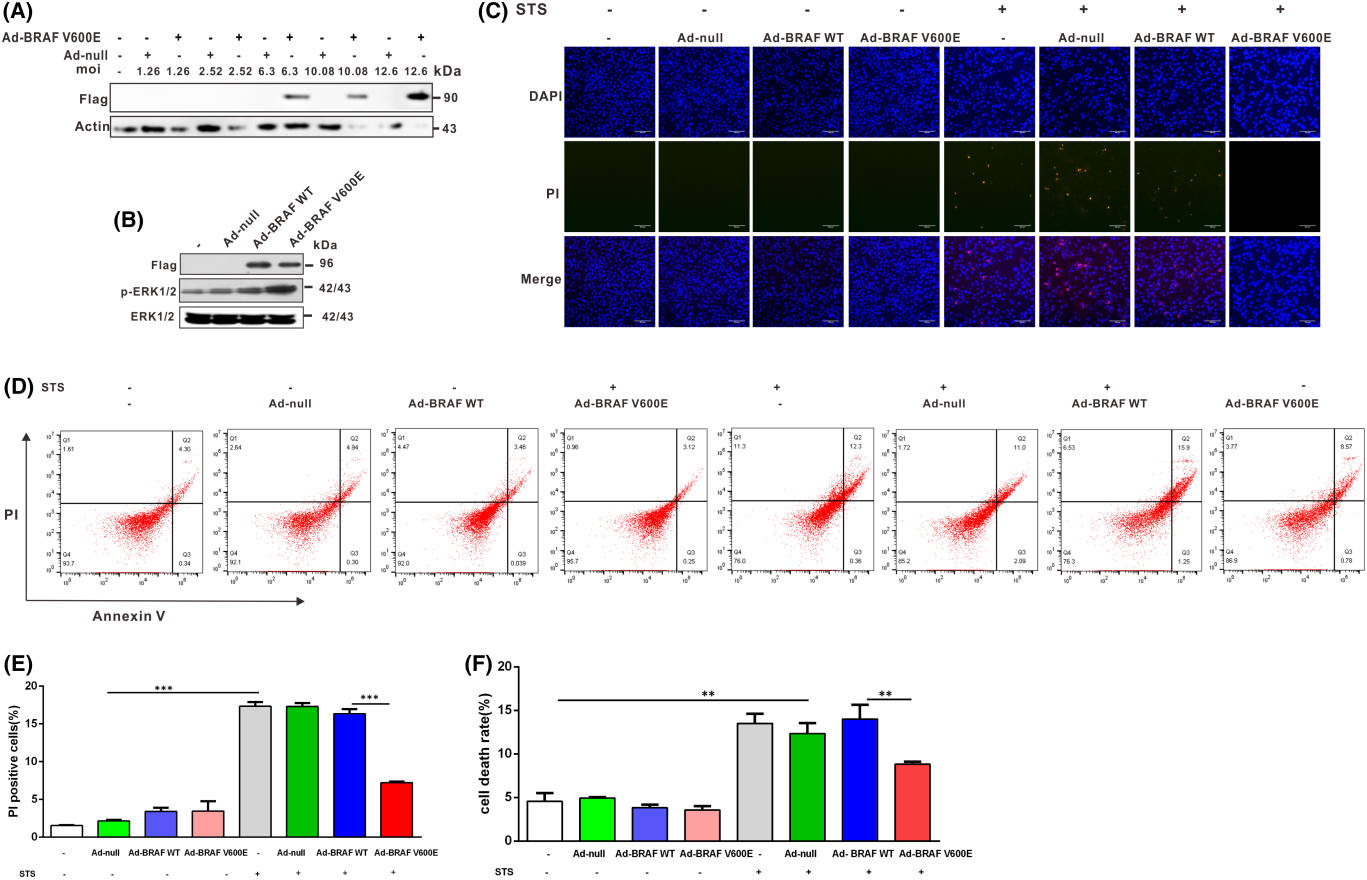
Adenovirus‐mediated overexpression of BRAF V600E in 8505C cells prevents STS‐induced cell death. (A) Multiplicity of infection (MOI) measurement of BRAF V600E overexpression adenovirus. 8505C cells were infected with adenoviruses overexpressing BRAF V600E at different MOI values (1.26, 2.52, 6.3, 10.08 and 12.6), and western blotting was used to detect the level of BRAF. β‐actin were used as a loading control. (B) 8505C cells were infected with adenoviruses overexpressing BRAF WT and BRAF V600E and western blotting was used to detect the Flag, phosphorylated and total ERK1/2 levels. (C) After infecting with adenoviruses overexpressing BRAF V600E and BRAF WT for 48 h, 8505C cells were treated with STS (200 nM). The 8505C cells were stained with PI (10 mg/ml) and DAPI, then observed under a fluorescence microscope. Scale bar, 200 μm. (D) 8505C cells were infected with adenoviruses overexpressing BRAF V600E and BRAF WT，the cell apoptosis and necrosis were assessed by Annexin V‐FITC/PI staining flow cytometry. (E) Two‐way anova followed by Bonferroni multiple comparisons test performed to statistical analyse of the proportion of the PI positive cells in each group of C. (F) Two‐way anova followed by Bonferroni multiple comparisons test was performed to statistical analyse the cell death rate of D. All results are presented as mean ± SD

To further explore the role of BRAF V600E in regulating death in papillary and anaplastic thyroid cancer cells, we constructed a BRAF WT and BRAF V600E overexpression adenovirus. 8505C cells were infected with adenoviruses overexpressing BRAF V600E at different MOIs, and western blotting was used to detect the BRAF level (Figure 4A). The results showed that BRAF WT and BRAF V600E were overexpressed in papillary and anaplastic thyroid cancer cells when infected with adenoviruses overexpressing BRAF WT and BRAF V600E (Figure 4B). Our results showed that the overexpression of BRAF V600E rather than BRAF WT reduced death of papillary and anaplastic thyroid cancer cells exposed to STS compared with the negative control group (Figures 3C,D and 4C,E). Nthy‐ori‐3.1 cells were also used as a control, and it was found that overexpression of BRAF V600E but not BRAF WT reduced death of Nthy‐ori‐3.1 after STS exposure compared to a negative control group (Figure 3E,F). The results of flow cytometry‐based measurement of apoptosis and necrosis using Annexin V‐FITC/PI staining further confirmed overexpression of BRAF V600E rather than BRAF WT attenuated death in papillary and anaplastic thyroid cancer cells exposed to STS (Figure 4D,F). Taken together, these results indicated that overexpression of BRAF V600E could attenuate papillary and anaplastic thyroid cancer cell death induced by STS exposure.

### 3.4 BRAF V600E inhibits mPTP opening induced by STS in papillary and anaplastic thyroid cancer cells

**FIGURE 5:**
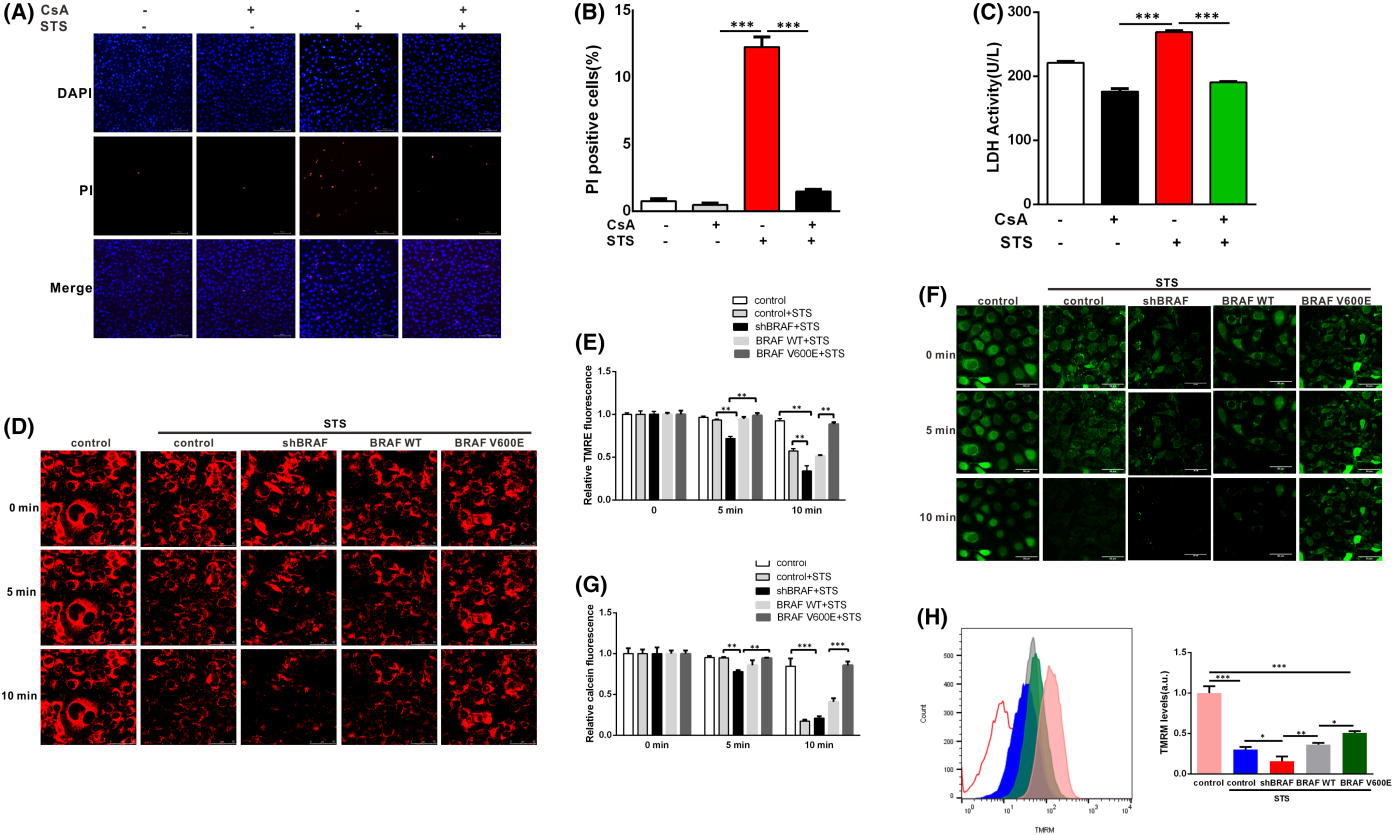
BRAF V600E prevents mPTP opening induced by STS in 8505C cells. (A) CsA (5 μM) treatment prevented STS‐induced cell death in 8505C cells exposed to 200 nM STS for 12 h compared with the control. Cell death was detected by PI assay. Scale bar, 500 μm. (B) The proportion of PI positive cells in each group was statistical analysed by two‐way anova followed by Bonferroni multiple comparisons test. *** indicated STS vs. CsA and STS + CsA. (C) 8505C cells were treated with CsA (5 μM) and STS (100 nM) for 24 h and the LDH activity (U/L) was measured. Statistical analysis was done by two‐way anova followed by Bonferroni multiple comparisons test. *** indicated STS vs. CsA and STS + CsA. (D) BRAF V600E overexpression delayed the loss of TMRE fluorescence in 8505C cells exposed to 200 nM STS compared to the control; Scale bar, 50 μm. (E) Two‐way anova followed by Bonferroni multiple comparisons test was performed to statistically analyse the intensity of the TMRE fluorescence in each group. **p < 0.01. (F) Forced expression of BRAF V600E delayed the loss of calcein fluorescence intensity in 8505C exposed to 200 nM STS compared to the control; scale bar, 50 μm. (G) Two‐way anova followed by Bonferroni multiple comparisons test was performed to statistically analyse the intensity of the calcein fluorescence in each group. **p < 0.01, ***p < 0.001. (H) The opening of mPTP channels in 8505C cells was detected by flow cytometry using TMRM. Two‐way anova followed by Bonferroni multiple comparisons test was performed to statistical analyse average fluorescence intensity after TMRM treatment. All results are presented as mean ± SD

We next tried to understand the mechanism of BRAF V600E inhibiting the death of papillary and anaplastic thyroid cancer cells. mPTP can be used as a target to prevent cell death in several pathological conditions, including cardiac ischaemia/reperfusion injury and diabetes. On the other hand, it could also block tumorigenesis by specifically inducing cell death. Therefore, mPTP plays an important role in the regulation of tumour cell death. We subsequently examined whether BRAF V600E‐based attenuation of STS‐induced papillary and anaplastic thyroid cancer cell death was due to the prevention of mPTP opening. Cyclosporin A (CsA) is a specific inhibitor of mPTP opening.^20^ First, we treated 8505C cells with CsA (5 μM), and then detected cell death by PI assay (Figure 5A,B) and LDH release assay (Figure 5C). We found that CsA (5 μM) treatment could also protect 8505C cells from cell death induced by STS (Figure 5A–C). These results suggest that STS promoted the death of 8505C cells by promoting the opening of mPTP.

Loss of the mitochondrial membrane potential (ΔΨm) promotes mPTP opening. TMRM (tetramethylrhodamine ethyl ester) is an indicator of mitochondrial membrane potential loss during necrosis and is widely used to indicate the level of death caused by ΔΨm loss.^21^, ^22^, ^23^ Subsequently, we investigated whether BRAF V600E is involved in the regulation of mPTP opening in papillary and anaplastic thyroid cancer cells after STS exposure. We successfully overexpressed and knockdown BRAF V600E in papillary and anaplastic thyroid cancer cells, as shown in Figures 2A and 4B. Then, 8505C and BCPAP cells were treated with TMRE (150 nM) and the opening of the mPTP was shown by rapid dissipation of TMRE fluorescence (Figures 5D and 7A). Compared with the negative control group and BRAF WT overexpression, the loss of TMRE was significantly delayed in papillary and anaplastic thyroid cancer cells with BRAF V600E overexpression (Figures 5D,E and 7A,C). Next, to further confirm the specificity of these events on pore dynamics, we used another established method to detect mPTP opening in intact cells.^24^, ^25^ Therefore, we incubated 8505C cells and BCPAP cells with calcein‐AM and cobalt‐chloride (CoCl_2_) to make the fluorescence localization of calcein in the mitochondria. The observed reduction in calcein fluorescence in the mitochondria determined the level of mPTP opening. Our results showed that compared with the negative control group, STS‐induced cell death in 8505C cells or BCPAP cells could lead to the loss of mitochondrial calcein fluorescence. Moreover, overexpression of BRAF V600E rather than BRAF WT in papillary and anaplastic thyroid cancer cells delayed the loss of calcein fluorescence after STS treatment (Figures 5F,G and 7B,D). Moreover, the fluorescence intensity of TMRM detected by flow cytometry further confirmed that BRAF V600E prevented the opening of mPTP channels induced by STS (Figures 5H and 7E). Taken together, these results demonstrate that the BRAF V600E mutant significantly inhibits mPTP opening in STS‐induced cell death.

### 3.5 BRAF V600E is located in mitochondria and inhibits mPTP opening by mediating the pERK‐pGSK‐CypD pathway in mitochondria

**FIGURE 6:**
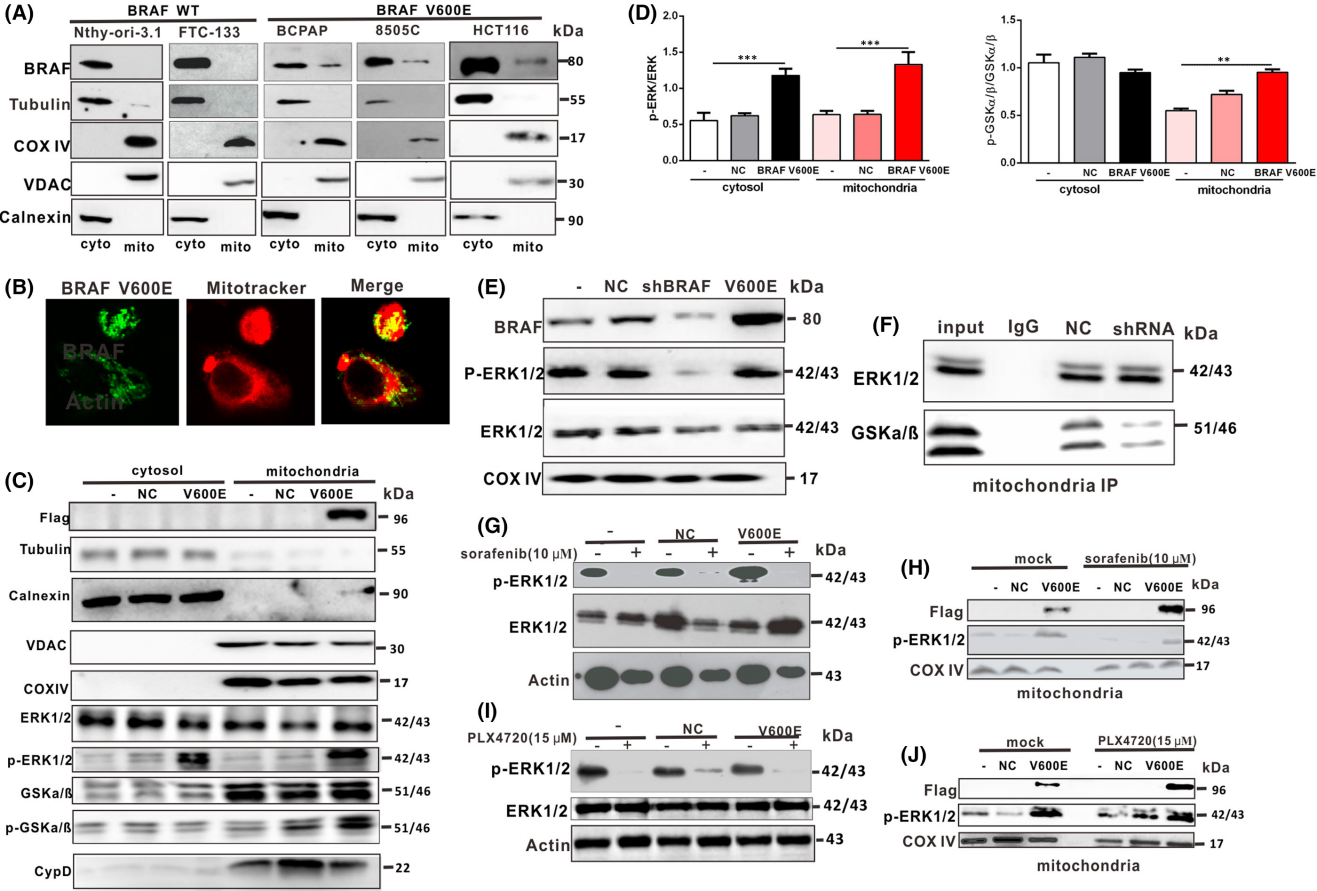
BRAF V600E mediates the p‐ERK‐p‐GSKα/β‐CypD pathway to prevent mPTP opening by interacting with mitochondrial ERK in 8505C cells. (A) Endogenous BRAF V600E was localized to the mitochondria. After Nthy‐ori‐3.1 thyroid cell (BRAF WT), FTC‐133 cell (BRAF WT), BCPAP cells (BRAF V600E mutant), 8505C cells (BRAF V600E mutant) and HCT116 human colon cancer cells (BRAF V600E mutant) were lysed, mitochondria were isolated by centrifugation. The localization of BRAF V600E and BRAF WT in mitochondria was detected by western blotting. Tubulin was used as a cytoplasmic marker, COX IV and VDAC were used as a mitochondrial marker. Calnexin as ER marker should be present at comparable levels in cytosol. (B) 8505C cells (BRAF V600E) were fixed and processed for immunofluorescence staining of BRAF (green fluorescence) and mitochondria (MitoTracker, red fluorescence). (C) 8505C cells were infected with adenoviruses overexpressing BRAF V600E，cytosol and mitochondria were isolated by centrifugation. Western blotting was used for detecting CypD, phosphorylated and total ERK1/2 and GSKα/β levels. Tubulin was used as a cytoplasmic marker, COX IV and VDAC as a mitochondrial marker. Calnexin as ER marker should be present at comparable levels in cytosol. (D) Statistical data showing the ratio of p‐ERK/ERK and p‐GSK/GSK of C. Statistical analysis was done by one‐way anova. ***p < 0.001 vs. control. (E) 8505C cells were infected with adenoviruses expressing BRAF V600E shRNA or overexpression and mitochondria were isolated by centrifugation. Western blotting was used for detecting phosphorylated and total ERK1/2 levels. COX IV as a mitochondrial marker. (F) Interaction between ERK1/2 and GSKα/β was detected by immunoprecipitation. 8505C cells were infected with adenoviruses expressing BRAF V600E shRNA, and mitochondria were isolated by centrifugation. Mitochondria were lysed and the interaction between ERK1/2 and GSKα/β were detected by immunoprecipitation and western blotting. (G) Nthy‐ori‐3.1 thyroid cells infected with adenoviruses overexpressing BRAF V600E and exposed to 10 μM sorafenib, the ratio of p‐ERK/ERK was measured by western blotting. (H) Nthy‐ori‐3.1 thyroid cells infected with adenoviruses overexpressing BRAF V600E and exposed to 10 μM sorafenib, the p‐ERK in mitochondria was measured by western blotting. (I) Nthy‐ori‐3.1 thyroid cells infected with adenoviruses overexpressing BRAF V600E and exposed to 15 μM PLX4720, the ratio of p‐ERK/ERK was measured by western blotting. (J) Nthy‐ori‐3.1 thyroid cells infected with adenoviruses overexpressing BRAF V600E and exposed to 15 μM PLX4720, the p‐ERK in mitochondria was measured by western blotting

It has previously been reported that BRAF V600E is located in the mitochondria.^15^ However, our experiment further proved that endogenous BRAF V600E is located in the mitochondria of both papillary and anaplastic thyroid cancer cells and colon cancer cells with the BRAF V600E mutation. Our results showed that in BCPAP (BRAF V600E mutant), 8505C cells (BRAF V600E mutant), and HCT116 colon cancer cells (BRAF V600E mutant), endogenous BRAF V600E was localized not only in the cytoplasm, but also in the mitochondria (Figure 6A). However, in Nthy‐ori‐3.1 (BRAF WT) thyroid cells and FTC‐133 (BRAF WT) thyroid cancer cells, the endogenous BRAF WT protein was localized only in the cytoplasm and not in mitochondria (Figure 6A). Moreover, 8505C cell (BRAF V600E) were fixed and processed for immunofluorescence staining of BRAF (green fluorescence) and mitochondria (MitoTracker, red fluorescence). As shown in Figure 6B, in 8505C cell, a significant portion of BRAF V600E colocalized with mitochondria.

**FIGURE 7:**
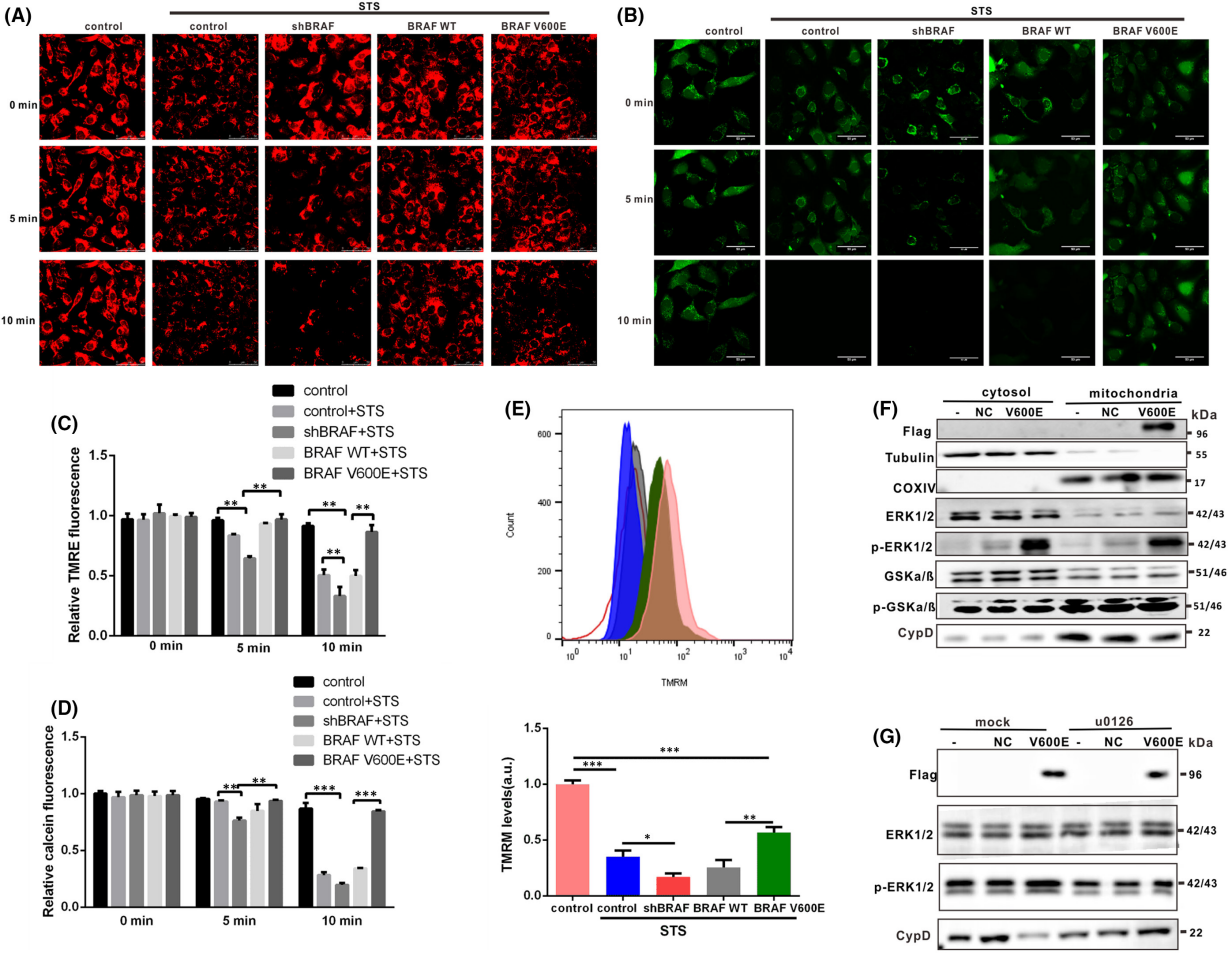
BRAF V600E prevents mPTP opening by p‐ERK‐p‐GSKα/β‐CypD pathway in BCPAP cells. (A) BRAF V600E overexpression delayed the loss of TMRE fluorescence in BCPAP cells exposed to 200 nM STS compared to the control; Scale bar, 50 μm. (B) Forced expression of BRAF V600E delayed the loss of calcein fluorescence intensity in BCPAP cell exposed to 200 nM STS compared to the control; scale bar, 50 μm. (C) Two‐way anova followed by Bonferroni multiple comparisons test was performed to statistical analyse the intensity of the TMRE fluorescence in each group. **p < 0.01. (D) Two‐way anova followed by Bonferroni multiple comparisons test was performed to statistical analyse the intensity of the calcein fluorescence in each group. **p < 0.01, ***p < 0.001. (E) The opening of mPTP channels in BCPAP cells was detected by flow cytometry. Two‐way anova followed by Bonferroni multiple comparisons test was performed to statistical analyse average fluorescence intensity after TMRM treatment. All results are presented as mean ± SD. (F) BCPAP cells were infected with adenoviruses overexpressing BRAF V600E, cytosol and mitochondria were isolated by centrifugation. Western blotting was used for detecting CypD, phosphorylated and total ERK1/2 and GSKα/β levels. Tubulin was used as a cytoplasmic marker, and COX IV as a mitochondrial marker. (G) BCPAP cells were infected with adenoviruses overexpressing BRAF V600E and u0126 treatment. Western blotting was used for detecting CypD, phosphorylated and total ERK1/2 of mitochondria isolated from BCPAP cells

Above results suggested that BRAF V600E was localized to the mitochondria and significantly inhibited mPTP opening during STS‐induced papillary and anaplastic thyroid cancer cell death. Next, we investigated the mechanism underlying the inhibition of mPTP opening with the BRAF V600E mutation. As shown in Figure 6C, overexpression of BRAF V600E promoted the phosphorylation of ERK in the mitochondria, and increased the level of p‐ERK/ERK (Figure 6C,D). The level of p‐ERK/ERK were detected in the mitochondria of BRAF V600E knockdown and overexpresssion. It was found that BRAF V600E knockdown decreased the level of p‐ERK/ERK, while overexpression promoted the level of p‐ERK/ERK in mitochondria (Figure 6E). Studies have shown that the activation of mitochondrial ERK in tumour cells desensitizes mPTP via the GSK and CypD signalling axis.^26^ GSK‐3 plays a key role in the regulation of the mPTP channel switch. Our results showed that levels of phosphorylated GSK were increased in papillary and anaplastic thyroid cancer cells overexpressing BRAF V600E (Figures 6C,D and 7F). As shown in Figure 6F, in the mitochondria of BRAF V600E mutant cells, there was an interaction between ERK1/2 and GSKa/ß, while upon BRAF V600E knockdown, interaction of GSKa/ß to ERK was decreased significantly. As the main regulator of mPTP, CypD can promote the opening of mPTP and induce cancer cells death.^27^, ^28^ We then investigated whether BRAF V600E inhibited mPTP opening and death by targeting the CypD protein. The results showed that BRAF V600E overexpression inhibited CypD expression (Figures 6C and 7F). Moreover, selective ERK inhibitor u0126 block mitochondrial ERK phosphorylation and rescued CypD level in papillary and anaplastic thyroid cancer cells (Figure 7G). Therefore, our study suggests that BRAF V600E constitutively activates mitochondrial ERK, thereby inhibiting GSK‐3‐dependent CypD phosphorylation, and thus making BRAF V600E mutant tumour cells more resistant to mPTP pore opening and subsequent cell death.

Sorafenib is an oral multi‐target TKI, which has activity on Raf kinase and Raf/MEK/ERK pathway (MAPK pathway). However, its clinical efficacy is limited, and it cannot completely inhibit the recurrence of melanoma and PTC with high BRAF mutation rate. Therefore, we speculate that BRAF mutation can inhibit the opening of mPTP pore and necrosis through pERK‐pGSK‐CypD pathway, which is one of the reasons that BRAF mutation affects the therapeutic effect of sorafenib. So we treated Nthy‐ori‐3.1 thyroid cells transiently overexpressing BRAF WT and BRAF V600E with 10 μM sorafenib for 24 h. We found that sorafenib inhibited p‐ERK/ERK in BRAF WT and BRAF V600E overexpressed cells (Figure 6G). At the same time, we extracted mitochondria and analysed the phosphorylation levels of ERK in the mitochondria of sorafenib‐treated and control groups by western blot. The results showed that sorafenib could not inhibit the phosphorylation of ERK in the mitochondria of BRAF V600E mutant cells and that the enhanced phosphorylation of mERK was not reversed by sorafenib (Figure 6H). To further investigate whether the enhancement of mERK phosphorylation is reversed by other BRAF V600E inhibitors, we treated cells with an effective and selective BRAF V600E inhibitor PLX4720 and found that BRAF V600E‐induced enhancement of mERK phosphorylation was also not reversed (Figure 6I,J). In conclusion, the phosphorylation of mitochondrial ERK is enhanced in BRAF V600E overexpressing cells, and this process was not inhibited by sorafenib and PLX4720.

## 4 DISCUSSION

BRAF mutations occur in many human cancers.^4^ Xing et al reported that knockdown of BRAF V600E inhibited the proliferation and growth of human PTC cells, as well as the tumorigenesis and tumour growth of PTC cells. These results suggest that BRAF V600E plays an important role in maintaining the proliferation, transformation and tumorigenicity of PTC cells carrying BRAF mutations, and that tumour growth from these cells continues to depend on BRAF V600E.^29^ It has been found that targeted expression of BRAF V600E in thyroid cells of transgenic mice can lead to dedifferentiation of PTC.^30^ In this study, we demonstrated another important pathway for conferring resistance to cell death in papillary and anaplastic thyroid cancer cells with the BRAF V600E mutation. We confirmed the mitochondrial localization of BRAF V600E and found for the first time that BRAF V600E mutant affects the interaction of mitochondrial ERK with GSKa/ß. In addition, BRAF V600E also promoted the phosphorylation of ERK in mitochondria and blocked mPTP through the pERK‐pGSK‐CypD pathway to resist cell death. Therefore, this study further elucidated the involvement of the BRAF V600E mutation in papillary and anaplastic thyroid cancer cells and provided a new intervention target for the BRAF V600E mutation in cancer. mPTPs are non‐selective channels located in the inner membrane of mitochondria.^31^ The opening of mPTP is the main event of the mitochondrial endogenous necrosis pathway, which causes mitochondrial permeability transition and mitochondrial potential loss.^32^, ^33^ The escape of apoptosis or antagonistic apoptosis of tumour cells is a key factor in their resistance to chemotherapy drugs. Some studies have shown that opening the mPTP could be a new method to increase or reverse multidrug resistance of tumour cells.^34^, ^35^, ^36^

Considering the potential role of mPTP in cancer, many researchers have studied the changes in mPTP activity and how mPTP regulatory mechanisms affect the occurrence and development of cancer.^37^, ^38^ Therefore, it is very important to study the regulation of mPTP opening in clinical cancer therapy strategies. GSK‐3 plays a key role in the regulation of the mPTP channel switch.^39^ It has two protein subtypes; GSK‐3α and GSK‐3β.^40^ GSK‐3β kinase is a negative regulator of mPTP, which has a significant regulatory effect on mPTP.^41^, ^42^, ^43^, ^44^ Phosphorylation of Ser 9 at the N‐terminus of GSK‐3β is the main mechanism underlying GSK‐3β.^45^ GSK‐3β directly phosphorylates Ser/Thr residues of CypD to regulate the opening and closing of mPTP.^46^ Phosphorylated CypD can combine with mPTP to promote pore opening.^47^ Whether in vitro or in vivo, it has been found that some inhibitors of PTP, such as CSA and its derivatives, play an important role in protecting cells from death.^48^, ^49^ Therefore, activation of GSK‐3β can increase the sensitivity of mPTP opening and inhibit tumour growth via this mechanism.

Because mitochondria play a key role in the regulation of cell death, the mitochondrial function of GSK‐3 is a particularly promising research field. Previous studies have shown that both ERK and GSK‐3β are partly located in the mitochondria and regulate mPTP opening via phosphorylation of GSK‐3β by mERK.^26^ In our study, we revealed for the first time that BRAF V600E promotes phosphorylation of ERK in papillary and anaplastic thyroid cancer mitochondria. We have also provided evidence that connects the BRAF V600E/mERK pathway and mPTP, and that in papillary and anaplastic thyroid cancer cell models, mitochondrial activation of ERK lead to desensitization of mPTP opening and increased resistance to death stimuli, which is of great significance for tumorigenesis. It has also been reported that mitochondrial constitutively activated ERK phosphorylates the serine residue of mGSK‐3β to inhibit its activity and thus hinder the phosphorylation of downstream CypD, triggering the closing of the PTP pore.^26^ In our experimental model, we hypothesized that BRAF V600E activated mERK and inhibited GSK‐3β activity, whereas GSK‐3β did not bind to CypD, instead phosphorylating CypD after inactivation, leading to PTP desensitization. Our research provided intensive study of the pathway of the BRAF V600E‐mERK‐GSK‐3β pathway regulating mPTP, and has thus identified a new selective target for anti‐tumour drugs that may restore the death threshold of tumour cells.

RAF inhibitors, such as sorafenib, are clinically used in the treatment of papillary and anaplastic thyroid cancer cells with BRAF mutations, but their efficacy is limited and cannot completely prevent the recurrence of advanced tumours, such as melanoma and PTC.^8^, ^9^ Furthermore, the application of MEK inhibitors in the treatment of advanced cancer has so far failed to achieve good clinical effect.^10^, ^11^ Therefore, it is necessary to find new targets for the treatment of papillary and anaplastic thyroid cancer cells with BRAF mutations. This may be an important outlet to study the regulatory mechanism of mitochondria‐located BRAF V600E. Our previous studies confirmed that BRAF V600E inhibits mitochondrial oxidative phosphorylation and promotes glycolysis through the HIF1α‐MYC‐PGC‐1β axis.^50^ While in this study, we further confirmed the mitochondrial localization of BRAF V600E and explained the molecular mechanism by which BRAF V600E is located in the mitochondria to resist cell death. In our study, we found that mitochondrial localization of BRAF V600E promotes the phosphorylation of mitochondrial ERK and the interaction of mERK between GSKa/ß, and mediates the regulation of mPTP via action on the pERK‐pGSK‐CypD pathway. Moreover, the phosphorylation level of ERK in the mitochondria of BRAF V600E overexpressing cells was not inhibited by sorafenib or PLX4720. Our study suggests that the regulation of mPTP in BRAF V600E mutant tumours may be an important therapeutic target. If we can inhibit the pathway of BRAF V600E regulating mPTP, it may have intervention and therapeutic effect on cancers with BRAF V600E mutations.

## AUTHOR CONTRIBUTIONS

**yanyan gao:** Conceptualization (equal); formal analysis (equal); funding acquisition (equal); project administration (equal); resources (equal); supervision (equal); writing – original draft (equal); writing – review and editing (equal). **deyu Zhang:** Methodology (equal); validation (equal). **fei Wang:** Investigation (supporting); methodology (supporting). **dejiu Zhang:** Data curation (equal); formal analysis (equal); methodology (equal). **Pei‐feng Li:** Methodology (equal); resources (equal); supervision (equal). **kun wang:** Data curation (equal); supervision (equal); writing – review and editing (equal).

## CONFLICT OF INTEREST

The authors declare no potential conflicts of interest.

## Data Availability

The data that support the findings of this study are available from the corresponding author upon reasonable request.
